# Adverse events related to bystander naloxone administration in cases of suspected opioid overdose in British Columbia: An observational study

**DOI:** 10.1371/journal.pone.0259126

**Published:** 2021-10-29

**Authors:** Amina Moustaqim-Barrette, Kristi Papamihali, Sierra Williams, Max Ferguson, Jessica Moe, Roy Purssell, Jane A. Buxton

**Affiliations:** 1 BC Centre for Disease Control, Vancouver, British Columbia, Canada; 2 Faculty of Medicine, Department of Emergency Medicine, University of British Columbia, Vancouver, British Columbia, Canada; 3 School of Population and Public Health, University of British Columbia, Vancouver, British Columbia, Canada; St. Michael’s Hospital, CANADA

## Abstract

**Introduction:**

Take-Home Naloxone programs have been introduced across North America in response to rising opioid overdose deaths. There is currently limited real-world data on bystander naloxone administration, overdose outcomes, and evidence related to adverse events following bystander naloxone administration.

**Methods:**

The research team used descriptive statistics from Take-Home Naloxone administration forms. We explored reported demographic variables and adverse events among people who received by-stander administered naloxone in a suspected opioid overdose event between August 31, 2012 and December 31, 2018 in British Columbia. We examined and contextualized differences across years given policy, program and drug toxicity changes. We used multivariate logistic regression to examine whether an association exists between number of ampoules of naloxone administered and the odds that the recipient will experience withdrawal symptoms.

**Results:**

A large majority (98.1%) of individuals who were administered naloxone survived their overdose and 69.2% had no or only mild withdrawal symptoms. Receiving three (Adjusted Odds Ratio (AOR) 1.64 (95% Confidence Interval (CI): 1.08–2.48)) or four or more (AOR 2.19 (95% CI: 1.32–3.62)) ampoules of naloxone was significantly associated with odds of moderate or severe withdrawal compared to receiving one ampoule of naloxone.

**Conclusions:**

This study provides evidence from thousands of bystander reversed opioid overdoses using Take-Home Naloxone kits in British Columbia, and suggests bystander-administered naloxone is safe and effective for opioid overdose reversal. Data suggests an emphasis on titration during bystander naloxone training in situations where the person experiencing overdose can be adequately ventilated may help avoid severe withdrawal symptoms. We identified a decreasing trend in the likelihood of moderate or severe withdrawal over the study period.

## Introduction

North America is currently experiencing a crisis of opioid overdoses and overdose deaths [1,2]. Since January 2016, over 16,000 people have died of opioid-related overdose in Canada [3]. While prescription opioids are also implicated, over 75% of Canadian overdose deaths involved the use of the illicit synthetic opioid fentanyl or its analogues [3]. In British Columbia (BC), the most heavily impacted Canadian province, a number of opioid overdose interventions have been introduced to curb rising deaths, including the establishment of overdose prevention services and supervised consumption sites (OPS/SCS), increasing access to opioid agonist therapy (OAT), and the ramp up of a provincial Take-Home Naloxone (THN) program [4]. Research suggests that the combined impact of these interventions have averted thousands of opioid overdose deaths in BC alone [5].

THN programs have been taken up widely in Canada [6]. Naloxone is a μ opioid receptor antagonist used reliably to reverse the life-threatening symptoms of opioid overdose [7]. As part of Canadian THN programs, individuals at risk of experiencing or witnessing an overdose are trained in appropriate overdose response and equipped with a THN kit. While intranasal formulations also exist as part of other territorial and provincial THN programs [6], and are available for First Nations peoples across Canada, the province of British Columbia publicly funds intramuscular injection [6]. The kits distributed by BC THN program include a carrying case, non-latex gloves, alcohol swab, a face shield with a one-way valve, three safety syringes, and three 1 mL naloxone ampoules (each ampoule contains 0.4 mg of naloxone and comes with an ampoule breaker to protect the hands of responders from cuts when snapping open the ampoule), as well as naloxone overdose response information forms (hereafter referred to as ‘administration forms’) and an instructional overdose response infographic.

A growing body of research has shown that naloxone is a safe and effective intervention for bystander opioid overdose reversal although its effects are time limited [8,9]. However, when naloxone is administered to individuals who are opioid-dependent, it can precipitate acute withdrawal syndrome which encompasses uncomfortable and often distressing symptoms including agitation, sweating, pain, vomiting, and flu-like symptoms, and rarely may cause more severe symptoms including pulmonary edema [10–13], seizure [14,15], and arrhythmias and cardiac arrest [16,17]. People who use drugs report experiencing extreme pain related to opioid withdrawal and health risks including receptive syringe sharing and overdose are associated with experiences of withdrawal [18,19].

While adverse outcomes following naloxone administration by health professionals have been found to be rare [20,21], there is little published research based on administrative data on bystander naloxone administration and the risk of opioid withdrawal. As part of the BC THN program, individuals are asked to complete and return an Overdose Response Information form to the BC Centre for Disease Control (BCCDC) after responding to an overdose. The present study uses data from the BC THN program to add to the literature regarding naloxone administration by bystanders to inform the increasing THN programs in North America, Australia, and Europe.

## Materials and methods

### 1.1. Data source

**Fig 1:**
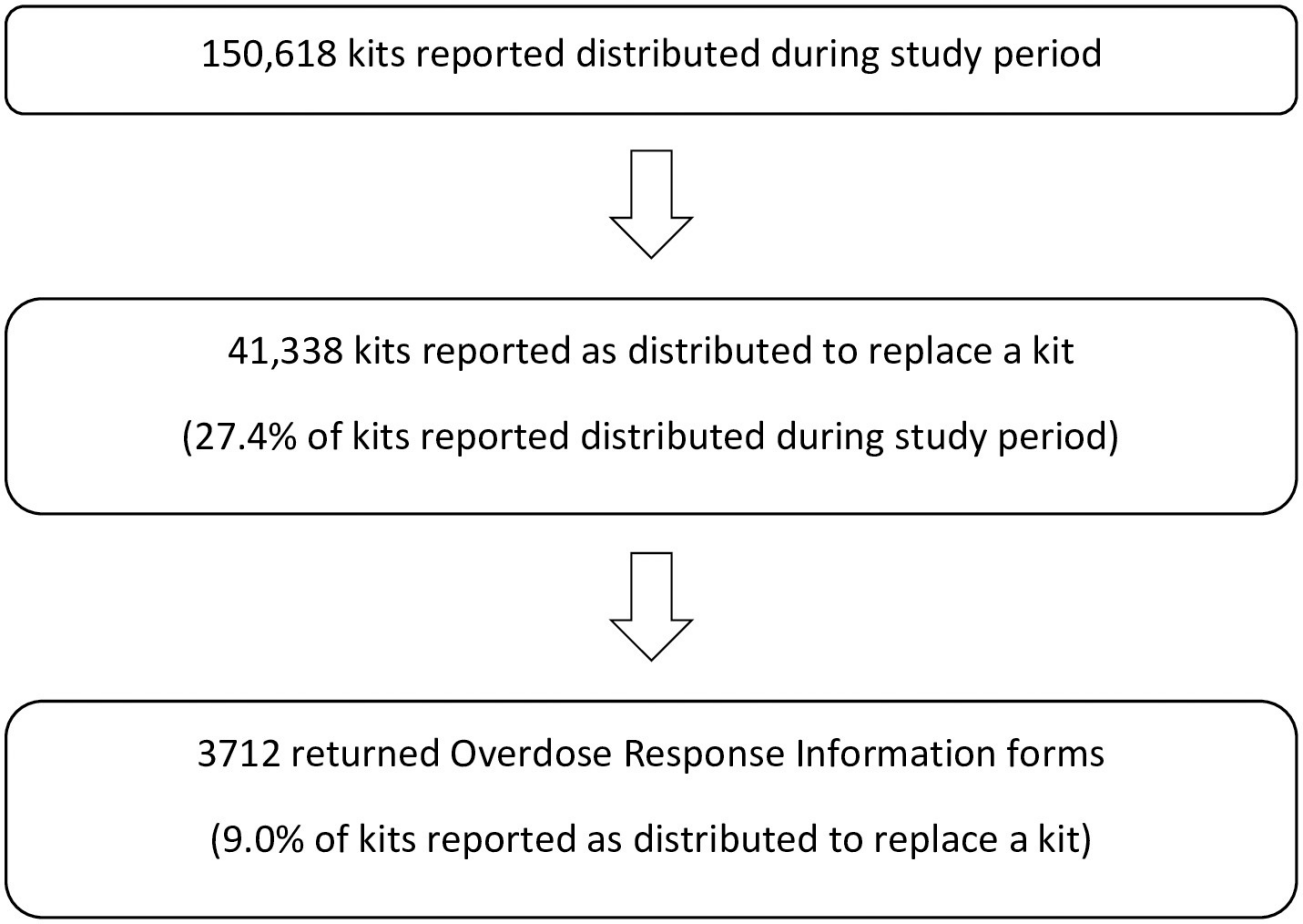


Since August 31, 2012, BCCDC has overseen a centralized THN program for the province of BC, with support from the BC Ministry of Health. THN kits have been available to individuals personally at risk of experiencing opioid overdose since program inception. The program was expanded to include those likely to witness an opioid overdose, such as family and friends, in September 2016. Training regarding overdose prevention, recognition and response including practice injection was provided at THN distribution sites. However, as training may be time consuming St. Paul’s Hospital emergency department providers, in collaboration with the BCCDC and Hello Cool World, spearheaded a brief online training module to support standardized training for individuals receiving a THN kit at a distribution site [22,23]. THN kits are available in a variety of locations including harm reduction and overdose prevention sites, shelters, health centres, treatment service centres, and community pharmacies and through peer outreach. A total of 150,618 naloxone kits were reported as distributed in the study timeframe, August 31^st^ 2012 to December 31^st^ 2018; of these 41,338 kits were reported as distributed to replace a kit which had been used to reverse an overdose; 9% of kits reported as used or 3712 returned forms comprised the study sample [24]. Fig 1 describes the proportion of returned forms relative to the number of kits distributed.

As part of the BC THN program, Overdose Response Information forms are available publicly online, at THN distributions sites, and are physically included in each THN kit for individuals to complete and return to the BCCDC after administering naloxone (See S1 Fig) [25,26]. We established validity of the Overdose Response Information form by consulting with Harm Reduction sites across the province and with people with lived and living experience of using drugs. Individuals can choose to fill and return the forms by email to the BCCDC directly, or complete and return forms to participating THN distribution sites, which in turn submit the forms to the BCCDC. Data collection through the administration form is anonymous, and information from paper forms is entered manually into a database at the BCCDC. This study utilizes the Overdose Response Information form data from program inception on August 31^st^ 2012 to December 31^st^ 2018. Please see supplementary materials for a copy of the form.

### 1.2. Study variables

The current study sought to understand the prevalence of adverse events after community naloxone administration in cases of suspected opioid overdose. The study participants who responded to the overdose rated the observed severity of withdrawal as ‘none, mild, moderate or severe’ based on their prior experience of withdrawal.

The research team created a binary outcome variable describing the prevalence of opioid withdrawal symptoms from the question “Did the person who received naloxone have any negative effects?” included in the administration form described above, where 0 = ‘None or mild withdrawal symptoms and 1 = ‘Moderate or severe withdrawal symptoms’. Withdrawal values were mutually exclusive.

The primary independent variable in this study was number of naloxone ampoules administered (1, 2, 3, 4 or more). Potential covariates reported by the overdose responder on the administration form included: age group of the individual who overdosed (under 19, 19–30, 31–60, over 60, unknown); gender of the individual who overdosed (male, female, trans and gender expansive [identified as either trans or other], unknown); year of overdose (before 2016, 2016, 2017, and 2018), which of BC’s five health regions the overdose occurred (Fraser Health, Interior Health, Island Health, Northern Health, and Vancouver Coastal Health); and the site of the overdose (private residence, street/alley/park, Single Room Occupancy Hotel [SRO]/supportive housing, shelter/tent, community agency/drop-in, and other settings [including hotel/motel], prefer not to say, don’t know, and ‘other’).

Year grouping was determined due to timing of change in number of naloxone ampoules in kits. At program inception each THN kit contained two naloxone ampoules. In response to the rise of ultra-potent synthetic opioids on the illicit market, and evidence suggesting a need for higher naloxone dosing [27,28], a third ampoule was added to the kits in March 2016. We determined the health region by recoding the city or community where the overdose occurred.

Other variables included whether the individual survived the overdose (yes, no, unknown), whether mouth-to-mouth rescue breathing was performed (yes, no, unknown), and whether emergency services (911) were called (yes, no, unknown).

### 1.3. Statistical analysis

We conducted all analyses using R version 3.5.3 [29]. We used descriptive statistics to describe characteristics of reported opioid overdoses in BC from THN program inception on August 31^st^ 2012 to December 31^st^ 2018. We also computed descriptive statistics for all covariates stratified by the main outcome variable, experiencing withdrawal effects. Subsequently, we used bivariable and multivariable logistic regression to examine correlates of experiencing withdrawal effects. In adherence with Hosmer and Lemeshow’s model building strategy [30], we included variables with a p-value < 0.25 in bivariable regression, and non-statistically significant but conceptually relevant covariates, in the multivariable regression model. We selected the final model using backwards selection and based on Akaike’s Information Criteria (AIC) [31]. Adjusted odds ratios (AOR) and 95% confidence intervals (CI). In the multivariable model, p-values under 0.05 are considered statistically significant.

### 1.4. Dealing with missing data

Complete cases analysis (CCA) was used for the primary analyses of this study. After examining patterns of missingness, missing observations were assumed to be missing at random and were recategorized as ‘unknown’ for all variables. Of 3712 administration forms available for the time period of interest, there were 488 records with missing information on age (13.1%), 380 on gender (10.2%), 414 on health region (11.2%), 370 on number of naloxone ampoules administered (10.0%), 1333 on withdrawal effects (36.0%), 578 on rescue breaths (15.6%), 180 related to calling emergency services (911) (4.8%), 608 on whether the individual survived the overdose (16.4%). For logistic regression analysis, unknown observations for the primary outcome variable (withdrawal effects) and independent variable (number of naloxone ampoules) were excluded, leaving 2027 in the analytical sample. Due to the high proportion of missingness associated with withdrawal symptoms, the final multivariable regression model was re-analysed using multiple imputation. Results were verified by running a parallel analysis using eight imputed datasets using multiple imputation by chained equation (MICE) [32].

### 1.5. Ethics

This study analysed anonymous administrative data. Ethics approval was obtained from the University of British Columbia Behavioural Ethics Board (H12-02557).

## Results

### 1.6. Overview of administration records

Table 1 presents summary characteristics of the total 3712 records included in this study stratified by year. Between 2012 and 2015 424 records (11.4% of the total) were returned, and 945 (25.5%), 1387 (37.4%), and 956 (25.8%) records returned in 2016, 2017, and 2018 respectively. Most overdoses where sociodemographic data are known occurred among men (69.1%) and those 31–60 years old (50.9%).

**Table 1:** Summary characteristics of Naloxone Administration Data, from program inception on August 31 2012 to December 31 2018.

Characteristics	Before 2016 (N = 424)	2016 (N = 945)	2017 (N = 1387)	2018 (N = 956)	Total (N = 3712)	p value
	n (%)	n (%)	n (%)	n (%)	n (%)	
**Gender**						< 0.001
Male	155 (36.6%)	583 (61.7%)	920 (66.3%)	648 (67.8%)	2306 (62.1%)	
Female	67 (15.8%)	281 (29.7%)	400 (28.8%)	272 (28.5%)	1020 (27.5%)	
Trans and Gender Expansive	0 (0.0%)	0 (0.0%)	0 (0.0%)	6 (0.6%)	6 (0.2%)	
Unknown	202 (47.6%)	81 (8.6%)	67 (4.8%)	30 (3.1%)	380 (10.2%)	
**Age Group**						< 0.001
Under 19	4 (0.9%)	9 (1.0%)	32 (2.3%)	29 (3.0%)	74 (2.0%)	
19–30	88 (20.8%)	296 (31.3%)	479 (34.5%)	341 (35.7%)	1204 (32.4%)	
31–60	153 (36.1%)	515 (54.5%)	733 (52.8%)	488 (51.0%)	1889 (50.9%)	
Over 60	8 (1.9%)	9 (1.0%)	24 (1.7%)	16 (1.7%)	57 (1.5%)	
Unknown	171 (40.3%)	116 (12.3%)	119 (8.6%)	82 (8.6%)	488 (13.1%)	
**Health Region**						< 0.001
Fraser Health	35 (8.3%)	326 (34.8%)	711 (51.3%)	226 (23.7%)	1298 (35.1%)	
Interior Health	27 (6.4%)	130 (13.9%)	221 (15.9%)	180 (18.8%)	558 (15.1%)	
Island Health	19 (4.5%)	98 (10.5%)	114 (8.2%)	63 (6.6%)	294 (8.0%)	
Northern Health	61 (14.5%)	232 (24.8%)	153 (11.0%)	152 (15.9%)	598 (16.2%)	
Vancouver Coastal Health	5 (1.2%)	50 (5.3%)	159 (11.5%)	322 (33.7%)	536 (14.5%)	
Unknown	273 (65.0%)	101 (10.8%)	28 (2.0%)	12 (1.3%)	414 (11.2%)	
**Number of ampoules administered**						< 0.001
1	208 (49.1%)	269 (28.5%)	314 (22.6%)	156 (16.3%)	947 (25.5%)	
2	171 (40.3%)	341 (36.1%)	494 (35.6%)	344 (36.0%)	1350 (36.4%)	
3	6 (1.4%)	164 (17.4%)	356 (25.7%)	208 (21.8%)	734 (19.8%)	
4 or more	6 (1.4%)	60 (6.3%)	158 (11.4%)	87 (9.1%)	311 (8.4%)	
Unknown	33 (7.8%)	111 (11.7%)	65 (4.7%)	161 (16.8%)	370 (10.0%)	
**Site of overdose**						< 0.001
Private residence	200 (47.2%)	329 (34.8%)	539 (38.9%)	283 (29.6%)	1351 (36.4%)	
Street/alley/park	94 (22.2%)	307 (32.5%)	420 (30.3%)	322 (33.7%)	1143 (30.8%)	
SRO/Supportive housing	59 (13.9%)	81 (8.6%)	75 (5.4%)	60 (6.3%)	275 (7.4%)	
Shelter/tent	35 (8.3%)	91 (9.6%)	166 (12.0%)	146 (15.3%)	438 (11.8%)	
Community agency/Drop-in	8 (1.9%)	37 (3.9%)	57 (4.1%)	43 (4.5%)	145 (3.9%)	
Other	28 (6.6%)	100 (10.6%)	130 (9.4%)	102 (10.7%)	360 (9.7%)	
**Adverse events**						< 0.001
None	121 (28.5%)	112 (11.9%)	604 (43.6%)	460 (48.3%)	1297 (35.0%)	
Mild withdrawal	30 (7.1%)	43 (4.6%)	166 (12.0%)	106 (11.1%)	345 (9.3%)	
Moderate withdrawal	5 (1.2%)	31 (3.3%)	151 (10.9%)	70 (7.3%)	257 (6.9%)	
Severe withdrawal	34 (8.0%)	82 (8.7%)	171 (12.4%)	29 (3.0%)	316 (8.5%)	
Other ^A^	19 (4.5%)	46 (4.9%)	59 (4.3%)	33 (3.5%)	157 (4.2%)	
Unknown	215 (50.7%)	630 (66.7%)	233 (16.8%)	255 (26.8%)	1333 (36.0%)	
**Rescue breathing performed**						< 0.001
No	244 (57.5%)	386 (40.8%)	476 (34.3%)	286 (29.9%)	1392 (37.5%)	
Yes	119 (28.1%)	388 (41.1%)	751 (54.1%)	484 (50.6%)	1742 (46.9%)	
Unknown	61 (14.4%)	171 (18.1%)	160 (11.5%)	186 (19.5%)	578 (15.6%)	
**911 called**						< 0.001
No	173 (40.8%)	348 (36.8%)	639 (46.1%)	352 (36.8%)	1512 (40.7%)	
Yes	241 (56.8%)	545 (57.7%)	664 (47.9%)	570 (59.6%)	2020 (54.4%)	
Unknown	10 (2.4%)	52 (5.5%)	84 (6.1%)	34 (3.6%)	180 (4.8%)	
**Survived the overdose**						<0.001
No	20 (4.7%)	9 (1.0%)	16 (1.2%)	13 (1.4%)	58 (1.6%)	
Yes	380 (89.6%)	782 (82.8%)	1252 (90.3%)	632 (66.1%)	3046 (82.1%)	
Unknown	24 (5.7%)	154 (16.3%)	119 (8.6%)	311 (32.5%)	608 (16.4%)	

^A^ Other effects included those who responded ‘don’t know’ or who reported that the individual who overdosed experienced feeling tired, hungry, shocked, grumpy, happy, frustrated, confused, and disoriented.

The vast majority of individuals administered naloxone during a suspected opioid overdose survived their overdose when outcomes were known (3046/3104, 98.1%). Where presence/absence of an adverse event was reported (n = 2372), most individuals (69.2%); 1642/2372) were reported by the responder to have experienced no or mild withdrawal effects; while 24.2% (573/2372) experienced moderate or severe withdrawal effects. ‘Other’ adverse events were reported experienced by 14.6% (345/2372) individuals, including those who responded ‘don’t know’ or who wrote additional comments on the survey reporting that the individual who overdosed felt tired, hungry, shocked, grumpy, happy, frustrated, confused, and disoriented. Where number of ampoules were reported, the proportion of respondents administering four or more ampoules initially rose each year (6/391, 1.5% before 2016, 60/834, 7.2% in 2016, 158/1322, 12.0% in 2017) before decreasing (87/795, 10.9%) in 2018.

Most overdoses occurred in private residences (1351/3712, 36.4%); the next most common setting reported for an overdose to occur was in a street, alley, or park (1143/3712, 30.8%). There was a significantly increasing trend of proportion of overdoses occurring in private residences demonstrated using a Cochran-Armitage test. Most cases (57.2%) reported that 911 was called (2020/3532) and rescue breathing was performed in 55.6% (1742/3134) of cases.

### 1.7. Characteristics of individuals who experienced withdrawal effects

Table 2 presents summary characteristics of the analytical sample, stratified by withdrawal effects. There were 2027 observations after removing ‘other’ adverse events and unknown observations. A slightly larger, non-significant proportion of males (13.6%) experienced moderate or severe withdrawal compared to females (10.2%). Of the health regions, Interior Health had the highest proportion of moderate or severe withdrawal (25.8%). Moderate or severe withdrawal symptoms increased with increasing number of naloxone ampoules administered; 9.8% (55/559) of individuals experienced moderate or severe withdrawal with one naloxone ampoule, compared with 20.5% (36/176) of those who received four or more ampoules.

**Table 2:** Summary characteristics of Naloxone Administration Data, from program inception on August 31 2012 to December 31 2018.

Characteristics	No or mild withdrawal symptoms (N = 1772)	Moderate or severe withdrawal symptoms (N = 255)	Total (N = 2027)	p-value
	n (%)	n (%)	n (%)	
**Gender**				0.16
Male	1098 (62%)	173 (67.8%)	1271 (62.7%)	
Female	520 (29.3%)	59 (23.1%)	579 (28.6%)	
Trans and Gender Expansive	6 (0.3%)	0 (0%)	6 (0.3%)	
Unknown	148 (8.4%)	23 (9%)	171 (8.4%)	
**Age Group**				0.39
Under 19	38 (2.1%)	2 (0.8%)	40 (2%)	
19–30	592 (33.4%)	80 (31.4%)	672 (33.2%)	
31–60	903 (51%)	143 (56.1%)	1046 (51.6%)	
Over 60	29 (1.6%)	3 (1.2%)	32 (1.6%)	
Unknown	210 (11.9%)	27 (10.6%)	237 (11.7%)	
**Year**				0.003
Before 2016	162 (9.1%)	23 (9%)	185 (9.1%)	
2016	196 (11.1%)	37 (14.5%)	233 (11.5%)	
2017	819 (46.2%)	138 (54.1%)	957 (47.2%)	
2018	595 (33.6%)	57 (22.4%)	652 (32.2%)	
**Health Region**				< 0.01
Fraser Health	652 (36.8%)	79 (31%)	731 (36.1%)	
Interior Health	233 (13.1%)	81 (31.8%)	314 (15.5%)	
Island Health	125 (7.1%)	14 (5.5%)	139 (6.9%)	
Northern Health	200 (11.3%)	23 (9%)	223 (11%)	
Vancouver Coastal Health	383 (21.6%)	34 (13.3%)	417 (20.6%)	
Unknown	179 (10.1%)	24 (9.4%)	203 (10%)	
**Number of ampoules**				< 0.01
1	504 (28.4%)	55 (21.6%)	559 (27.6%)	
2	726 (41%)	96 (37.6%)	822 (40.6%)	
3	402 (22.7%)	68 (26.7%)	470 (23.2%)	
4 or more	140 (7.9%)	36 (14.1%)	176 (8.7%)	
**Site of overdose**				0.01
Private Residence	672 (37.9%)	126 (49.4%)	798 (39.4%)	
Street/Alley/Park	525 (29.6%)	64 (25.1%)	589 (29.1%)	
SRO/Supportive Housing	115 (6.5%)	16 (6.3%)	131 (6.5%)	
Shelter/Tent	219 (12.4%)	24 (9.4%)	243 (12%)	
Community Agency/Drop-in	86 (4.9%)	6 (2.4%)	92 (4.5%)	
Other ^A^	155 (8.7%)	19 (7.5%)	174 (8.6%)	
**911 called**				0.21
No	788 (44.5%)	128 (50.2%)	916 (45.2%)	
Yes	928 (52.4%)	121 (47.5%)	1049 (51.8%)	
Unknown	56 (3.2%)	6 (2.4%)	62 (3.1%)	
**Rescue breathing performed**				0.197
No	709 (40%)	87 (34.1%)	796 (39.3%)	
Yes	975 (55%)	154 (60.4%)	1129 (55.7%)	
Unknown	88 (5%)	14 (5.5%)	102 (5%)	

^A^ ‘Other’ includes hotels/motels, prefer not to say, don’t know, and ‘other’.

The proportion of moderate or severe withdrawal symptoms across sites of overdose did not differ significantly; the highest proportion (15.8%) occurring among those who overdosed in a private residence, and the lowest (6.5%) among those who overdosed in a community agency or drop-in centre. In overdose events for which rescue breathing was performed, 13.6% of cases experienced moderate or severe withdrawal, compared with 10.9% who did not receive rescue breathing.

### 1.8. Correlates of experiencing moderate or severe withdrawal symptoms with naloxone administration

In the multivariable logistic regression model (Table 3), there was an increasing trend in the odds of experiencing moderate or severe withdrawal associated with increasing ampoules of naloxone administered compared to those who had received one ampoule of naloxone (AOR = 1.29; 95% CI: 0.89–1.88 for two ampoules, AOR = 1.64; 95% CI: 1.08–2.48 for three ampoules, and AOR = 2.19; 95% CI: 1.32–3.62 for four or more ampoules). Conversely, there was a decreasing trend in the odds of moderate or severe withdrawal over time, compared to the years before 2016 although these results were not significant. The association was statistically significant when comparing the odds of three or four or more ampoules to a single administered ampoule; the association of two ampoules was non-significant compared to one ampoule. There was no significant association between any gender or age group and the likelihood of moderate or severe withdrawal. Compared to Vancouver Coastal Health, those who experienced an overdose in Interior Health had three times the odds of experiencing moderate or severe withdrawal (AOR = 3.16; 95% CI: 1.88–5.33). Analyses conducted with the imputed data (S1 Table) showed associations were consistent with the non-imputed data. A similarly increasing trend in the odds of experiencing moderate or severe withdrawal associated with increasing ampoules of naloxone administered compared to those who had received one ampoule of naloxone was observed (AOR = 1.09; 95% CI: 0.20–3.90 for two ampoules, AOR = 1.31; 95% CI: 1.04–1.64 for three ampoules, and AOR = 1.77; 95% CI: 1.31–2.38 for four or more ampoules).

**Table 3:** Main effects. Adjusted odds ratios and 95% confidence intervals for experiencing moderate or severe withdrawal symptoms. (n = 2027).

*Main Effects Model*	AOR (95% CI)	P-value
**Number of ampoules**		
1	1.00	-
2	1.29 (0.89–1.88)	0.17
3	1.64 (1.08–2.48)	0.02
4 or more	2.19 (1.32–3.62)	<0.01
**Year**		
Before 2016	1.00	-
2016	1.13 (0.55–2.32)	0.74
2017	0.92 (0.45–1.90)	0.82
2018	0.47 (0.22–1.02)	0.06
**Gender**		
Male	1.00	-
Female, Trans and Gender Expansive	0.75 (0.54–1.04)	0.08
Unknown	1.09 (0.55–2.13)	0.81
**Age Group**		
Under 19	0.35 (0.08–1.54)	0.17
19–30	0.85 (0.63–1.16)	0.30
31–60	1.00	-
Over 60	0.62 (0.18 = 2.11)	0.44
Unknown	0.78 (0.45–1.35)	0.38
**Health Region**		
Fraser Health	0.90 (0.53–1.51)	0.68
Interior Health	3.16 (1.88–5.33)	<0.01
Island Health	0.99 (0.48–2.03)	0.99
Northern Health	0.98 (0.54–1.78)	0.96
Vancouver Coastal Health	1.00	-
Unknown	1.13 (0.52–2.44)	0.76
**Rescue breathing performed**		
No	1.00	-
Yes	1.38 (1.01–1.87)	0.04
Unknown	1.16 (0.61–2.19)	0.65
**911 called**		
No	1.00	-
Yes	0.77 (0.58–1.03)	0.08
Unknown	0.54 (0.22–1.34)	0.19

Abbreviations: AOR, Adjusted odds ratio; CI, confidence interval.

## Discussion

This study sought to examine the prevalence of adverse events associated with the BC THN program since program inception on August 31, 2012, to the end of 2018, and to examine correlates of experiencing moderate or severe withdrawal. We found over 98% of individuals who were administered naloxone to reverse an overdose in BC survived the overdose event, and most individuals (69.2%) had no or only mild withdrawal symptoms. The study found that receiving three (AOR 1.64 (CI: 1.08–2.48)), or four or more (AOR 2.19 (CI: 1.32–3.62)) ampoules of naloxone was associated with increased odds of moderate or severe withdrawal compared to receiving one ampoule of naloxone. We also found a non-significant decreasing trend in the odds of experiencing moderate or severe withdrawal symptoms over time.

To our knowledge, this is the first study examining the prevalence of adverse events associated with THN programs in Canada. Our findings are consistent with earlier studies from the United States and Europe [8,33–36]. In a systematic review McDonald and Strang consolidated data from five studies that did not contain duplicate samples and found that a survival rate associated with THN of 99.1% (20 confirmed deaths per 2336 naloxone administrations) [37]. The survival rate identified by McDonald and Strang was similar to that identified in our study; an important consideration for our data is that it is unclear how many people were found unresponsive and received naloxone after they were already deceased. McDonald and Strang also consolidated data on adverse events, and report 65 instances of withdrawal symptoms for the 2336 naloxone administrations included a rate of 2.8%. A recent systematic review by Moe et al. 2020 [27] found that 11% of individuals experienced withdrawal, and 1% experienced pulmonary edema, after naloxone administration in community settings or emergency departments. Our study reported a higher estimate compared to Moe et al. and McDonald & Strang; 15.4% of the sample reported moderate or severe withdrawal [27,37]. The estimates of withdrawal by Moe et al. and McDonald & Strang were generated by systematic reviews. McDonald & Strang calculated their estimate based off articles ranging from 2001 to 2014 and in countries including the United States, Canada, the United Kingdom, and Germany, while Moe et al calculated their estimate based off articles identified in their larger systematic review ranging from 1972 to 2018 from studies based in North America and Europe. The differences in location and timeframe reasonably explain the difference in estimate of withdrawal prevalence.

In most BC health regions, the number of naloxone ampoules administered increased over time. Nevertheless, while fentanyl became more common in the illicit drug supply and the amount of naloxone administered increased over time, the proportion of individuals experiencing moderate or severe withdrawal did not increase (14.4% in 2017 vs 8.7% in 2018). This may be indicative of improved overdose response in the community, increased overdose awareness, and/or increased availability and accessibility of training materials. Importantly, overdose response training emphasizing titration of doses has been shown to lessen withdrawal symptoms and the experience and confidence of responders may have increased over time [13,38].

After adjustment for covariates, we found evidence of higher moderate or severe withdrawal with the administration of four or more naloxone ampoules which aligns with previous research on withdrawal associated with high dose naloxone [13]. The vast majority of naloxone kits were distributed after the introduction of a third ampoule; however, even after the introduction of a third dose of naloxone in March 2016, most cases continued to use one to two doses of naloxone to reverse an overdose (808/1322 (61.1%) in 2017 and 500/795 (62.9%) in 2018). Nevertheless, we found a consistent increasing trend in naloxone ampoules administered over time, which we believe to be associated with the emergence of fentanyl and its analogues in BC.

A systematic review by Moe et al. evaluated the relationship between naloxone dose and overdose reversal/adverse events before and after the emergence of ultra-potent opioids and provides evidence to support a potential association of an increase trend of naloxone ampoules administered and the emergence of fentanyl and its analogues [27]. Moe et al. found among patients with presumed exposure to fentanyl or ultra-potent opioids, 56.9% responded to an initial naloxone dose ≤0.4 mg, compared with 80.2% of people who used heroin [39].

The illicit drug supply is constantly changing. Since 2020, benzodiazepines have been increasingly identified in the opioid supply [34]. While naloxone may successfully reverse the respiratory depression of opioid overdose, individuals may remain unconscious due to non-opioid sedatives, including benzodiazepines and etizolam. Continued sedation may result in responders administering more naloxone and precipitating withdrawal. Vomiting is a symptom of opioid withdrawal associated with naloxone administration and may compromise an individual’s airway if they remain unconscious. Understanding how to lessen the severity of withdrawal following naloxone administration, including using titration, is increasingly important [13,34]. Public health surveillance of opioid through drug checking [40], and enforcement samples [41] of the drug supply changes allows for responsiveness and flexibility in harm reduction interventions and education [42].

Our study shows that the vast majority of overdoses were successfully reversed using THN with no or only mild withdrawal symptoms and suggest that a low dose of naloxone should be administered initially and additional doses titrated until adequate reversal of respiratory depression is achieved, as long as ventilation can be supported, in order to avoid precipitating opioid withdrawal [13,38]. Administration of four or more ampoules of naloxone were associated with withdrawal symptoms, but on occasions are necessary with the increasing toxic drug supply. Titrating doses may require more in-depth in-person training for bystanders, and should be coupled with other actions, including providing ventilation/breaths to prevent respiratory arrest and hypoxemia. In 2021 the U.S. Food and Drug Administration approved an intranasal nasal spray which delivers 8mg compared to the previously approved 2mg and 4mg doses; high dose naloxone disallows titration of doses within the community setting and increases the risk of precipitating withdrawal for recipients [13,43,44].

Our study has some key limitations. Data collected relied upon individuals responding to an overdose completing the Overdose Response Information form and on their understanding of adverse events and withdrawal symptoms, and these data are susceptible to recall (inaccurate or incomplete recollections) and reporting bias. While most people with THN kits use opioids and are familiar with withdrawal symptoms and severity, the outcome variable of withdrawal being reported by the naloxone administrator introduces the risk of bias to the study [45]. Due to the low-barrier and voluntary nature of THN program reporting, we do not know how these statistics compare to overdoses for which no administration form was completed and this limits the generalizability of these results. A total of 150,618 kits were reported distributed during the study period; of these 41,338 were reported distributed to replace a kit that had been used to reverse an overdose. We analysed 3712 returned forms thus our data represents 9.0% of the kits reported as used. This low response rate means we cannot generalize findings to the larger population of people who use THN kits. We also cannot quantify how many distinct individuals these data represent (individuals may have been administered naloxone more than once and responders may be reporting more than one administration event), though we do not feel that this changes the interpretation of the results, as discrete overdose events each have unique characteristics. We also do not know the final outcome i.e. survival for individuals who were transported by ambulance. Nevertheless, our data provides valuable insight into the THN program, as experimental data on this subject is largely precluded due to the logistical and ethical issues related to conducting randomised experimental trials in patients at risk of dying from opioid overdose.

This study offers important insights into experiences of people who receive naloxone through the BC THN program. A large majority of the those who were administered naloxone to reverse an overdose, over 98%, survived the overdose event. Most experienced no or mild withdrawal symptoms (69%). The prevalence of people experiencing moderate or severe withdrawal symptoms after receiving naloxone was 15%, while a decreasing trend of withdrawal symptoms throughout the study period was identified.

THN administration in BC is safe and effective to reverse opioid overdose. An emphasis on titrating doses of naloxone where the person experiencing overdose can be adequately ventilated may help avoid moderate or severe withdrawal symptoms.

## Supporting information

S1 Fig Overdose Response Information form page 1. (TIFF)

S2 Fig Overdose Response Information form page 2. (TIFF)

S1 Table Adjusted odds ratios and 95% confidence intervals for experiencing moderate or severe withdrawal symptoms with multiply imputed dataset (Multiple Imputation by Chained Equation (MICE)). (DOCX)

## References

[1] Fischer B, Keates A, Bühringer G, Reimer J, Rehm J. Non-medical use of prescription opioids and prescription opioid-related harms: why so markedly higher in North America compared to the rest of the world? Addict Abingdon Engl. 2014 Feb;109(2):177–81. doi: 10.1111/add.12224 23692335

[2] Guy GP, Zhang K, Bohm MK, Losby J, Lewis B, Young R, et al. Vital Signs: Changes in Opioid Prescribing in the United States, 2006–2015. MMWR Morb Mortal Wkly Rep. 2017 Jul 7;66(26):697–704. doi: 10.15585/mmwr.mm6626a4 28683056 PMC5726238

[3] Health Canada. Opioid-related Harms in Canada [Internet]. Government of Canada. 2020 [cited 2020 Apr 9]. https://health-infobase.canada.ca/substance-related-harms/opioids/.

[4] Young S, Williams S, Otterstatter M, Lee J, Buxton J. Lessons learned from ramping up a Canadian Take Home Naloxone programme during a public health emergency: a mixed-methods study. BMJ Open. 2019;9(10):e030046. doi: 10.1136/bmjopen-2019-030046 31662368 PMC6830612

[5] Irvine MA, Kuo M, Buxton JA, Balshaw R, Otterstatter M, Macdougall L, et al. Modelling the combined impact of interventions in averting deaths during a synthetic-opioid overdose epidemic. Addict Abingdon Engl. 2019;114(9):1602–13. doi: 10.1111/add.14664 31166621 PMC6684858

[6] Amina Moustaqim-Barrette, Tara Elton-Marshall, Pamela Leece, Carole Morissette, Katherine Rittenbach, Jane Buxton. Environmental Scan Naloxone Access and Distribution in Canada. 2019 Jun 30; https://open.library.ubc.ca/media/stream/pdf/52383/1.0379400/5.

[7] Ngai SH, Berkowitz BA, Yang JC, Hempstead J, Spector S. Pharmacokinetics of naloxone in rats and in man: basis for its potency and short duration of action. Anesthesiology. 1976 May;44(5):398–401. doi: 10.1097/00000542-197605000-00008 1267205

[8] McDonald R, Strang J. Are take-home naloxone programmes effective? Systematic review utilizing application of the Bradford Hill criteria. Addict Abingdon Engl. 2016 Mar 30;111(7):1177–87. doi: 10.1111/add.13326 27028542 PMC5071734

[9] Giglio RE, Li G, DiMaggio CJ. Effectiveness of bystander naloxone administration and overdose education programs: a meta-analysis. Inj Epidemiol. 2015;2(1):10. doi: 10.1186/s40621-015-0041-8 27747742 PMC5005759

[10] Nath SS, Tripathi M, Pandey C, Rao B. Naloxone-induced pulmonary edema: a potential cause of postoperative morbidity in laparoscopic donor nephrectomy. Indian J Med Sci. 2009;63(2):72–5. doi: 10.4103/0019-5359.49240 19359770

[11] Reed CR, Glauser FL. Drug-induced noncardiogenic pulmonary edema. Chest. 1991 Oct;100(4):1120–4. doi: 10.1378/chest.100.4.1120 1914570

[12] Sporer KA, Dorn E. Heroin-related noncardiogenic pulmonary edema: a case series. Chest. 2001 Nov;120(5):1628–32. doi: 10.1378/chest.120.5.1628 11713145

[13] Purssell R, Godwin J, Moe J, Buxton J, Crabtree A, Kestler A, et al. Comparison of rates of opioid withdrawal symptoms and reversal of opioid toxicity in patients treated with two naloxone dosing regimens: a retrospective cohort study. Clin Toxicol. 2020 May 13;1–9. doi: 10.1080/15563650.2020.1758325 32401548

[14] Kim HK, Nelson LS. Reducing the harm of opioid overdose with the safe use of naloxone: a pharmacologic review. Expert Opin Drug Saf. 2015;14(7):1137–46. doi: 10.1517/14740338.2015.1037274 25865597

[15] Kerr D, Kelly A-M, Dietze P, Jolley D, Barger B. Randomized controlled trial comparing the effectiveness and safety of intranasal and intramuscular naloxone for the treatment of suspected heroin overdose. Addict Abingdon Engl. 2009;104(12):2067–74. doi: 10.1111/j.1360-0443.2009.02724.x 19922572

[16] Cuss FM, Colaço CB, Baron JH. Cardiac arrest after reversal of effects of opiates with naloxone. Br Med J Clin Res Ed. 1984 Feb 4;288(6414):363–4. doi: 10.1136/bmj.288.6414.363 6419929 PMC1444229

[17] Wermeling DP. Review of naloxone safety for opioid overdose: practical considerations for new technology and expanded public access. Ther Adv Drug Saf. 2015;6(1):20–31. doi: 10.1177/2042098614564776 25642320 PMC4308412

[18] Bluthenthal RN, Simpson K, Ceasar RC, Zhao J, Wenger L, Kral AH. Opioid withdrawal symptoms, frequency, and pain characteristics as correlates of health risk among people who inject drugs. Drug Alcohol Depend. 2020 Jun;211:107932. doi: 10.1016/j.drugalcdep.2020.107932 32199668 PMC7259345

[19] Coffin PO, Tracy M, Bucciarelli A, Ompad D, Vlahov D, Galea S. Identifying Injection Drug Users at Risk of Nonfatal Overdose. Acad Emerg Med. 2007;14(7):616–23. doi: 10.1197/j.aem.2007.04.005 17554010

[20] Kelly A-M, Kerr D, Dietze P, Patrick I, Walker T, Koutsogiannis Z. Randomised trial of intranasal versus intramuscular naloxone in prehospital treatment for suspected opioid overdose. Med J Aust. 2005;182(1):24–7. doi: 10.5694/j.1326-5377.2005.tb06550.x 15651944

[21] Loimer N, Hofmann P, Chaudhry HR. Nasal administration of naloxone is as effective as the intravenous route in opiate addicts. Int J Addict. 1994 Apr;29(6):819–27. doi: 10.3109/10826089409047912 8034388

[22] BC Centre for Disease Control. Training & Resources [Internet]. Toward the heart. 2020 [cited 2020 May 19]. https://towardtheheart.com/naloxone-training.

[23] New naloxone training app helps teach people how to save lives [Internet]. 2017 [cited 2021 Jul 9]. https://towardtheheart.com/update/naloxone-training-app-launches.

[24] BC Take Home Naloxone Program [Internet]. BC Centre for Disease Control; 2021 Jun [cited 2021 Jul 15]. https://towardtheheart.com/assets/uploads/1624385842yo4HOUJqNp8Qj85OBaAQMA8VhmDkiKs8iI8g7bZ.pdf.

[25] Toward the Heart, BC Centre for Disease Control (BCCDC). Take Home Naloxone: Overdose Response Information Form [Internet]. Toward the Heart; 2018. https://towardtheheart.com/assets/uploads/15456830156WfBfJTnliUOffTNY2O5YN6lYH2W98GdgBsB1WT.pdf.

[26] Take Home Naloxone Sites [Internet]. Towards the Heart. [cited 2021 Sep 16]. https://towardtheheart.com/thn-sites.

[27] Moe J, Godwin J, Purssell R, O’Sullivan F, Hau JP, Purssell E, et al. Naloxone dosing in the era of ultra-potent opioid overdoses: a systematic review. CJEM. 2020;22(2):178–86. doi: 10.1017/cem.2019.471 31955714

[28] Connors NJ, Nelson LS. The Evolution of Recommended Naloxone Dosing for Opioid Overdose by Medical Specialty. J Med Toxicol Off J Am Coll Med Toxicol. 2016 Sep;12(3):276–81. 10.1007/s13181-016-0559-3 PMC4996792 27271032

[29] The R Foundation. R: The R Project for Statistical Computing [Internet]. 2019 [cited 2019 Mar 26]. https://www.r-project.org/.

[30] Hosmer David W., Lemeshow Stanley, Sturdivant Rodney X. Model-Building Strategies and Methods for Logistic Regression. In: Applied Logistic Regression [Internet]. John Wiley & Sons, Ltd; 2013 [cited 2019 Mar 26]. p. 89–151. https://onlinelibrary.wiley.com/doi/abs/10.1002/9781118548387.ch4.

[31] step function | R Documentation [Internet]. [cited 2020 Sep 10]. https://www.rdocumentation.org/packages/stats/versions/3.6.2/topics/step.

[32] Azur MJ, Stuart EA, Frangakis C, Leaf PJ. Multiple imputation by chained equations: what is it and how does it work? Int J Methods Psychiatr Res. 2011 Feb 24;20(1):40–9. doi: 10.1002/mpr.329 21499542 PMC3074241

[33] Enteen L, Bauer J, McLean R, Wheeler E, Huriaux E, Kral AH, et al. Overdose prevention and naloxone prescription for opioid users in San Francisco. J Urban Health Bull N Y Acad Med. 2010;87(6):931–41. doi: 10.1007/s11524-010-9495-8 20967505 PMC3005091

[34] Rowe C, Santos G-M, Vittinghoff E, Wheeler E, Davidson P, Coffin PO. Predictors of participant engagement and naloxone utilization in a community-based naloxone distribution program. Addict Abingdon Engl. 2015;110(8):1301–10. 10.1111/add.12961 PMC4503489 25917125

[35] Bennett AS, Bell A, Tomedi L, Hulsey EG, Kral AH. Characteristics of an overdose prevention, response, and naloxone distribution program in Pittsburgh and Allegheny County, Pennsylvania. J Urban Health Bull N Y Acad Med. 2011;88(6):1020–30. 10.1007/s11524-011-9600-7 PMC3232410 21773877

[36] Maxwell S, Bigg D, Stanczykiewicz K, Carlberg-Racich S. Prescribing naloxone to actively injecting heroin users: a program to reduce heroin overdose deaths. J Addict Dis. 2006;25(3):89–96. doi: 10.1300/J069v25n03_11 16956873

[37] McDonald R, Strang J. Are take-home naloxone programmes effective? Systematic review utilizing application of the Bradford Hill criteria. Addiction. 2016;111(7):1177–87. doi: 10.1111/add.13326 27028542 PMC5071734

[38] Purssell R, Buxton JA, Godwin J, Moe J. Potent sedatives in opioids in BC: Implications for resuscitation, and benzodiazepine and etizolam withdrawal | British Columbia Medical Journal. BC Med J. 2021 May;63(4):177–8.

[39] Moe J, Godwin J, Purssell R, Hau JP, Purssell E, Curran J, et al. Naloxone dosing in the era of ultra-potent opioid overdoses: a systematic review.: 9. 10.1017/cem.2019.471 31955714

[40] Long V, Tobias S, Lysyshyn M, Sage C, Bridgerman J, Gibson E, et al. A Report on British Columbia’s Unregulated Drug Supply: Results from British Columbia’s Community Drug Checking Service, June 2018 –December 2019 [Internet]. Vancouver: BC Centre on Substance Use; 2020 [cited 2021 Sep 16]. https://drugcheckingbc.ca/wp-content/uploads/sites/2/2020/12/BCCSU_BCs_Drug_Checking_Results_Report.pdf.

[41] Canada H. Drug Analysis Service [Internet]. 2021 [cited 2021 Sep 16]. https://www.canada.ca/en/health-canada/services/health-concerns/controlled-substances-precursor-chemicals/drug-analysis-service.html.

[42] BC Centre for Disease Control Fact Sheet: Etizolam in British Columbia’s Illicit Drug Market [Internet]. BC Centre for Disease Control; 2021 Mar. https://towardtheheart.com/assets/uploads/1620768097sIdcTgyTPH64olUECbSHJYRMklJdFBZngLCwls5.pdf.

[43] Commissioner O of the. FDA Approves Higher Dosage of Naloxone Nasal Spray to Treat Opioid Overdose [Internet]. FDA. FDA; 2021 [cited 2021 Sep 16]. https://www.fda.gov/news-events/press-announcements/fda-approves-higher-dosage-naloxone-nasal-spray-treat-opioid-overdose.

[44] Hill LG, Zagorski CM, Loera LJ. Increasingly powerful opioid antagonists are not necessary. Int J Drug Policy. 2022 Jan;99:103457. 10.1016/j.drugpo.2021.103457 PMC8454200 34560623

[45] Moustaqim-Barrette A, Papamihali K, Crabtree A, Graham B, Karamouzian M, Buxton JA. Correlates of take-home naloxone kit possession among people who use drugs in British Columbia: A cross-sectional analysis. Drug Alcohol Depend. 2019 Dec;205:107609. doi: 10.1016/j.drugalcdep.2019.107609 31654839

