# Supplementary figures and images for: Adverse events related to bystander naloxone administration in cases of suspected opioid overdose in British Columbia: An observational study
Source: PLoS One. 2021 Oct 29;16(10):e0259126. doi: 10.1371/journal.pone.0259126 (PMC8555799; doi:10.1371/journal.pone.0259126)

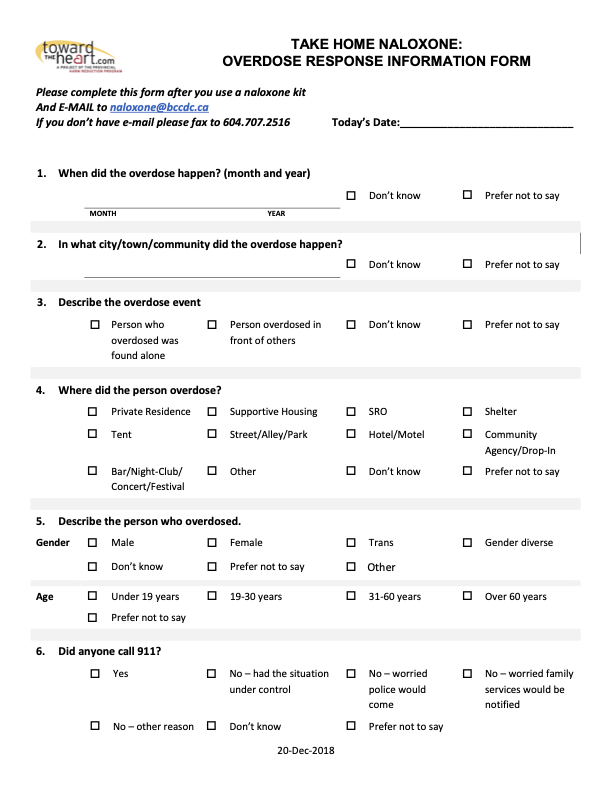

Supplement: S1 Fig — (TIFF) [file pone.0259126.s001.TIFF]

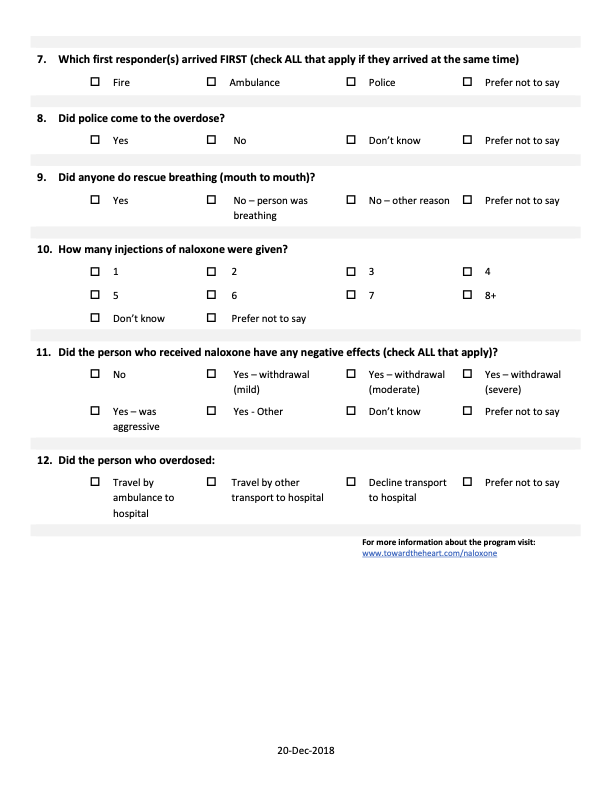

Supplement: S2 Fig — (TIFF) [file pone.0259126.s002.TIFF]
