# Supplementary material for: Adverse events related to bystander naloxone administration in cases of suspected opioid overdose in British Columbia: An observational study
Source: PLoS One. 2021 Oct 29;16(10):e0259126. doi: 10.1371/journal.pone.0259126 (PMC8555799; doi:10.1371/journal.pone.0259126)
Supplement: S1 Table — (DOCX) [file pone.0259126.s003.docx]

| **Supplementary Table 1 – Adjusted odds ratios and 95% confidence intervals for experiencing moderate or severe withdrawal symptoms with multiply imputed dataset (Multiple Imputation by Chained Equation (MICE)).** | | |
| --- | --- | --- |
|  |  | |
| *Main Effects Model* | AOR (95% CI) | P-value |
|  |  |  |
| **Number of ampoules** |  |  |
| 1 | 1.00 | - |
| 2 | 1.09 (0.20 – 3.90) | 0.42 |
| 3 | 1.31 (1.04 – 1.64) | 0.02 |
| 4 or more | 1.77 (1.31 – 2.38) | <0.01 |
| **Year** |  |  |
| Before 2016 | 1.00 | - |
| 2016 | 2.44 (1.82 – 3.38) | <0.01 |
| 2017 | 1.61 (1.20 – 2.17) | <0.01 |
| 2018 | 0.67 (0.48 – 9.30) | 0.02 |
| **Gender** |  |  |
| Male | 1.00 | - |
| Female, Trans and Gender Expansive | 1.07 (0.90 – 1.27) | 0.44 |
| **Age Group** |  |  |
| Under 19 | 0.48 (0.24 – 9.30) | 0.03 |
| 19 - 30 | 0.97 (0.82 – 1.14) | 0.73 |
| 31 - 60 | 1.00 | - |
| Over 60 | 0.97 (0.52 – 1.78) | 0.92 |
| **Health Region** |  |  |
| Fraser Health | 0.63 (0.51 – 7.90) | <0.01 |
| Interior Health | 1.25 (0.97 – 1.60) | 0.09 |
| Island Health | 0.93 (0.68 – 1.26) | 0.63 |
| Northern Health | 0.59 (0.44 – 7.91) | <0.01 |
| Vancouver Coastal Health | 1.00 | - |
| **Rescue breathing performed** |  |  |
| No | 1.00 | - |
| Yes | 1.23 (1.04 – 1.45) | 0.02 |
| **911 called** |  |  |
| No | 1.00 | - |
| Yes | 0.85 (0.72 – 1.00) | 0.05 |
| *N= 3422*  *Abbreviations: AOR, Adjusted odds ratio; CI, confidence interval*  **Sample size too small for accurate effect estimate*  ^A ‘^Other’ includes hotels/motels, prefer not to say, don’t know, and ‘other’ | | |
